# Research Progress on Polyphenols and Polysaccharides from Marine Seaweeds: Promising Diabetes Management Natural Products

**DOI:** 10.3390/md24060208

**Published:** 2026-06-11

**Authors:** Yiqiao Wang, Zhiyu Lin, Haiying Zhang, Yanan Gao, Yan Liu, Jingwei Liang

**Affiliations:** 1 Engineering Research Center of Tropical Medicine Innovation and Transformation of Ministry of Education, School of Pharmacy, Hainan Medical University, Haikou 571199, China; wangyiqiao@muhn.edu.cn (Y.W.); kevin120407@outlook.com (Z.L.); zhyhymu@163.com (H.Z.); 2 Hainan Pharmaceutical Research and Development Science Park, Hainan Medical University, Haikou 571199, China; 3 School of Pharmacy, China Medical University, Shenyang 110122, China

**Keywords:** marine seaweed, polyphenols, phlorotannins, polysaccharides, fucoidan, type 2 diabetes mellitus, α-glucosidase inhibition, insulin resistance, structure–activity relationship, antioxidant

## Abstract

Type 2 diabetes mellitus (T2DM) is a major global health burden characterized by insulin resistance, progressive pancreatic β-cell dysfunction, and chronic metabolic dysregulation. Marine seaweeds have emerged as a valuable source of bioactive natural products, particularly polyphenols and polysaccharides, with promising potential for diabetes management. This review focuses on three major contributions: first, the structural diversity of seaweed-derived polyphenols and polysaccharides; second, their multi-target mechanisms of glucose regulation; and third, the structure–activity relationships governing their bioactivities. Current evidence shows that these compounds may help manage type 2 diabetes in several ways, including inhibition of α-amylase and α-glucosidase, attenuation of oxidative stress and chronic inflammation, enhancement of insulin secretion and insulin sensitivity, regulation of lipid metabolism, and modulation of gut microbiota. Key structural determinants such as degree of polymerization, hydroxyl group density, sulfation level, molecular weight, and chemical modifications are discussed in relation to their functional properties. By linking chemical structure with biological function, these findings highlight marine seaweeds as a rich reservoir of multi-target therapeutic candidates for T2DM management and provide a scientific basis for their development as functional food ingredients or lead compounds for novel diabetes management drugs.

## 1. Introduction

Diabetes mellitus, particularly type 2 diabetes mellitus (T2DM), represents one of the most prevalent and rapidly growing chronic metabolic disorders worldwide, affecting over 500 million individuals globally and imposing an enormous socioeconomic burden [1]. T2DM is characterized by persistent hyperglycemia resulting from the interplay of insulin resistance in peripheral tissues and the progressive decline of pancreatic β-cell function. Multiple pathological factors—including chronic low-grade inflammation, oxidative stress, lipotoxicity, and gut microbiota dysbiosis—collectively contribute to its complex metabolic network of disturbances [2,3].

Marine seaweeds (macroalgae) have attracted increasing scientific attention as a rich and structurally diverse source of bioactive natural products. Unlike terrestrial plants, seaweeds are continuously exposed to high salinity, intense UV radiation, and complex ecological pressures, which have driven the evolution of unique classes of polyphenols and polysaccharides with distinctive structural features and broad biological activities [4,5]. Among them, seaweed-derived polyphenols—particularly phlorotannins exclusively found in brown algae—and polysaccharides such as fucoidan, alginate, carrageenan, laminarin, and ulvan have demonstrated significant potential for diabetes management in numerous preclinical studies [6].

The glucose-regulating mechanisms of these marine bioactives are multifaceted and complementary, encompassing inhibition of carbohydrate-digesting enzymes (α-amylase and α-glucosidase), attenuation of oxidative stress and chronic inflammation, enhancement of insulin secretion and sensitivity, regulation of lipid metabolism, and modulation of gut microbiota [7]. Furthermore, their biological activities are closely governed by structural features, including the degree of polymerization and hydroxylation (for polyphenols), and the degree of sulfation, molecular weight, monosaccharide composition, and chemical modifications (methylation/acetylation) for polysaccharides. Despite the growing body of preclinical evidence, a comprehensive review integrating the structural characterization, glucose-regulating mechanisms, and structure–activity relationships of both seaweed polyphenols and polysaccharides is currently lacking. This review aims to systematically summarize recent advances in these areas, with the goal of providing a scientific basis for the development of marine seaweed-derived compounds as functional food ingredients or lead compounds for novel therapeutics for diabetes management.

To ensure a comprehensive and focused synthesis, a structured literature search was conducted using PubMed, Web of Science and Google Scholar. The search covered studies published from 2015 to 2026, with the final search updated on Marth 2026. Search terms were organized around three major themes: marine seaweed sources, bioactive compound classes, and diabetes-related outcomes. The main search strategy combined the following terms: “marine seaweed”, “macroalgae”, “brown algae”, “red algae”, “green algae”, “polyphenol”, “phlorotannin”, “polysaccharide”, “fucoidan”, “alginate”, “carrageenan”, “laminarin”, “ulvan”, “type 2 diabetes”, “hyperglycemia”, “insulin resistance”, “glucose metabolism”, “α-glucosidase”, “α-amylase”, “oxidative stress”, “inflammation”, and “gut microbiota”. Studies were included if they investigated seaweed-derived polyphenols, phlorotannins, polysaccharides, or related derivatives and reported glucose-regulating or metabolism-related effects. Studies unrelated to marine seaweeds, lacking diabetes-related outcomes, or providing insufficient information on compound source, experimental model, or mechanism were excluded. Due to the heterogeneity of compound structures, experimental models, and outcome measures, the included evidence was synthesized narratively according to compound category, biological mechanism, and structure–activity relationship.

## 2. Seaweed-Derived Polyphenols and Diabetes

Seaweed polyphenols represent a structurally diverse and biologically active class of natural products derived from marine macroalgae, exhibiting unique structural features and bioactivities that differ substantially from those of terrestrial plant polyphenols. These differences are primarily driven by the unique ecological niche and adaptive strategies of marine algae, including exposure to high salinity, intense UV radiation, and complex ecological pressures.

### 2.1. Overview of Seaweed Polyphenols

#### Sources and Classification of Seaweed Polyphenols

**Figure 1:**
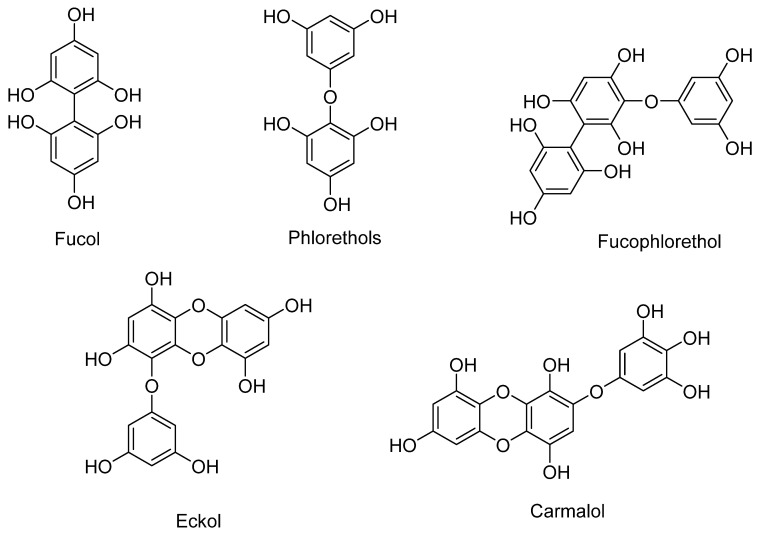
Chemical structures of representative seaweed polyphenols. Representative phlorotannin oligomers with varying degrees of polymerization and linkage types, including Fucol, Phlorethols, Fucophlorethol, Eckol and Carmalol. Structural diversity underlies the broad spectrum of biological activities.

Seaweed polyphenols represent a structurally diverse group of bioactive compounds predominantly found in marine macroalgae, especially brown seaweeds. The most extensively studied class is phlorotannins, which are polymers of phloroglucinol (1,3,5-trihydroxybenzene) units unique to brown algae (Phaeophyceae). These compounds exhibit a wide range of polymerization degrees, resulting in molecules with varying molecular weights and structural complexities. Phlorotannins can be classified into several subclasses based on the type of linkages between phloroglucinol units, including fucols (aryl-aryl bonds), phlorethols (aryl-ether bonds), fucophlorethols (mixed linkages), eckols, and carmalols, each conferring distinct chemical properties and bioactivities (Figure 1) [1,8]. Besides phlorotannins, other polyphenolic compounds in seaweeds include bromophenols, flavonoids, phenolic terpenoids, and mycosporine-like amino acids [8,9].

Structurally, phlorotannins possess multiple hydroxyl groups, and their antioxidant capacity is achieved through the scavenging of free radicals. Their polymeric nature allows for interactions with other seaweed components such as polysaccharides, which can influence their solubility, stability, and bioavailability [10]. The chemical complexity of phlorotannins poses challenges for extraction and quantification, necessitating advanced analytical techniques such as nuclear magnetic resonance (NMR), mass spectrometry, and Fourier-transform infrared spectroscopy (FTIR) for accurate structural elucidation [11,12].

The biofunctional properties of seaweed polyphenols are closely related to their structural features. For instance, the degree of polymerization and specific linkages in phlorotannins influence their ability to inhibit enzymes like α-amylase and α-glucosidase, relevant for glucose-regulating effects [11]. Moreover, molecular docking studies have identified key interactions of major phlorotannins such as dieckol and phlorofucofuroeckol-A with G protein-coupled receptors (GPCRs), suggesting mechanisms underlying their pharmacological activities [13]. Despite their promising bioactivities, the poor solubility, instability, and extensive metabolism of phlorotannins limit their bioavailability, necessitating advanced delivery strategies [14].

### 2.2. Glucose-Regulating Effects of Seaweed Polyphenols

Type 2 diabetes mellitus (T2DM) is a chronic metabolic disorder characterized by persistent hyperglycemia, with its core pathophysiological features involving insulin resistance and the progressive decline of pancreatic β-cell function. During disease progression, insulin sensitivity in peripheral tissues—including the liver, skeletal muscle, and adipose tissue—is markedly reduced, leading to impaired glucose uptake and utilization, accompanied by increased hepatic glucose production. Multiple factors, including chronic low-grade inflammation, oxidative stress, lipotoxicity, and gut microbiota dysbiosis, collectively contribute to the development of a complex network of metabolic disturbances.

Accumulating preclinical evidence has demonstrated that seaweed-derived polyphenols exert significant glucose-regulating and metabolism-modulating effects across multiple levels. These effects are not only reflected in the alleviation of diabetic phenotypes in animal models but are also supported by mechanistic insights at both cellular and molecular levels. The relevant findings are summarized in Table 1.

#### 2.2.1. Antioxidative Stress

Oxidative stress is widely recognized as a common driving force in the onset and progression of T2DM. Under chronic hyperglycemic and lipotoxic conditions, multiple processes—including mitochondrial electron transport chain overload, activation of NADPH oxidases, and accumulation of advanced glycation end products (AGEs)—collectively amplify the generation of reactive oxygen species (ROS) [15]. This persistent oxidative burden contributes to pancreatic β-cell dysfunction, impaired insulin signaling in peripheral tissues, and the establishment of a positive feedback loop with chronic low-grade inflammation.

Seaweed-derived polyphenols—particularly phlorotannins from brown algae—exhibit potent, multi-level antioxidant activities, largely attributed to their high density of phenolic hydroxyl groups and their ability to stabilize radical intermediates. These compounds can directly scavenge free radicals (e.g., DPPH and ABTS) and inhibit lipid peroxidation, leading to decreased intracellular ROS and malondialdehyde (MDA) levels [16]. They also enhance endogenous antioxidant defense systems by upregulating key enzymes such as superoxide dismutase (SOD), catalase (CAT), and glutathione peroxidase (GPx), thereby alleviating cellular oxidative stress under hyperglycemic or oxidative conditions [17].

Importantly, by attenuating oxidative stress, seaweed polyphenols can suppress inflammation amplification and restore insulin signaling homeostasis, thereby providing mechanistic support for their potential in diabetes management [18]. Targeting oxidative stress is therefore a critical strategy for improving the pancreatic β-cell microenvironment and maintaining glucose homeostasis.

#### 2.2.2. Promotion of Insulin Secretion

Seaweed-derived polyphenols have been shown to enhance insulin secretion and improve glycemic control in T2DM by modulating multiple molecular targets involved in pancreatic β-cell function. Polyphenol-rich extracts from *Undaria pinnatifida* demonstrated significant improvements in glucose metabolism and restoration of islet integrity in diabetic mouse models. These effects were partly attributed to the upregulation of key genes associated with insulin signaling pathways, including PI3K, Akt, AMPK, and GLUT4, alongside the downregulation of negative regulators such as FOXO1 and GSK-3β [19].

In addition, seaweed polyphenols exert protective effects against oxidative stress and inflammation in β-cells. Activation of AMP-activated protein kinase (AMPK) by seaweed polyphenols promotes insulin secretion and glucose uptake by improving cellular energy metabolism and alleviating lipotoxicity. Collectively, seaweed polyphenols target multiple pathways involved in insulin secretion and β-cell preservation, highlighting their potential as natural therapeutic agents for improving insulin dynamics and reducing hyperglycemia [19,23].

#### 2.2.3. Upregulation of Insulin Receptor Expression

The upregulation of insulin receptor (IR) expression represents a key mechanism by which seaweed-derived polyphenols exert their insulin-sensitizing and glucose-regulating effects. Polyphenols extracted from marine algae, such as *Undaria pinnatifida*, can increase the expression of insulin receptor substrates and downstream signaling molecules, including PI3K and Akt, which play central roles in the insulin signaling cascade [19]. Upregulation of these downstream signaling components may be mediated, at least in part, through the suppression of inflammatory mediators such as nitric oxide (NO) and prostaglandin E_2_ (PGE_2_), as well as the inhibition of cyclooxygenase-2 (COX-2) [20].

This coordinated regulation enhances insulin binding and signal transduction efficiency, thereby promoting the translocation of glucose transporter type 4 (GLUT4) to the plasma membrane and increasing glucose uptake in peripheral tissues. By restoring or enhancing insulin receptor expression and function, seaweed polyphenols help overcome insulin resistance—a hallmark of T2DM—thereby contributing to improved glycemic control and a reduced risk of diabetes-related complications [24].

#### 2.2.4. Regulation of Lipid Metabolism

Seaweed-derived polyphenols contribute to metabolic regulation in T2DM by modulating lipid metabolism, which is closely linked to insulin sensitivity and glucose homeostasis. Dyslipidemia—characterized by elevated triglycerides, reduced HDL-C, and increased free fatty acids—exacerbates insulin resistance and β-cell dysfunction in T2DM. Polyphenolic compounds from brown algae improve lipid profiles by regulating key enzymes and pathways involved in lipid synthesis, oxidation, and transport.

Mechanistically, seaweed polyphenols can activate AMP-activated protein kinase (AMPK), a central regulator of cellular energy metabolism. AMPK activation promotes fatty acid oxidation while inhibiting lipogenesis, thereby reducing ectopic lipid accumulation in metabolic tissues such as the liver and skeletal muscle [19,23]. Studies on *Ishige okamurae* have demonstrated that its polyphenolic constituents can significantly suppress lipid accumulation in 3T3-L1 preadipocytes [21]. By targeting both lipid metabolism and inflammation, seaweed polyphenols mitigate key pathogenic factors underlying T2DM progression, highlighting their potential as promising adjunctive agents for integrated T2DM management.

#### 2.2.5. Inhibition of Carbohydrate-Digesting Enzymes

Seaweed-derived polyphenols exhibit potent inhibitory effects on key carbohydrate-digesting enzymes, namely α-amylase and α-glucosidase, both of which play critical roles in starch hydrolysis and postprandial glucose regulation. Extracts from brown seaweeds such as *Durvillaea antarctica*, *Silvetia compressa*, and *Ecklonia arborea* have been reported to contain high levels of phlorotannins and sulfated polysaccharides associated with pronounced inhibitory effects on both enzymes [22,25].

Mechanistically, the inhibitory effects are attributed to the ability of polyphenolic functional groups to interact with the active or allosteric sites of these enzymes, thereby reducing catalytic efficiency and slowing carbohydrate digestion. This delay in glucose release not only decreases the rate of glucose absorption but also mitigates postprandial glucose spikes, which are closely associated with insulin resistance and β-cell dysfunction. These studies underscore the therapeutic potential of seaweed-derived polyphenols as natural inhibitors of α-amylase and α-glucosidase for the management of postprandial hyperglycemia [23,25].

### 2.3. Structure–Activity Relationships of Seaweed Polyphenols

**Figure 2:**
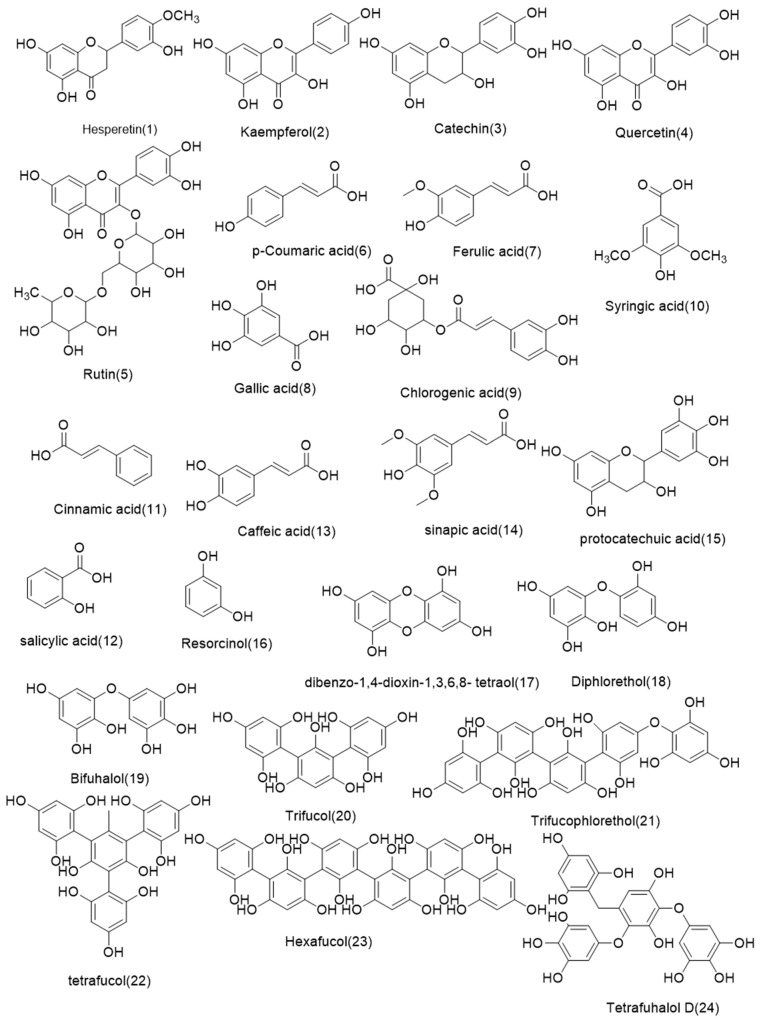
Chemical structures of representative seaweed-derived polyphenolic compounds discussed in this review.These compounds represent structurally diverse classes of seaweed polyphenols, including flavonoids, phenolic acids, simple phenols, and phlorotannins.

**Figure 3:**
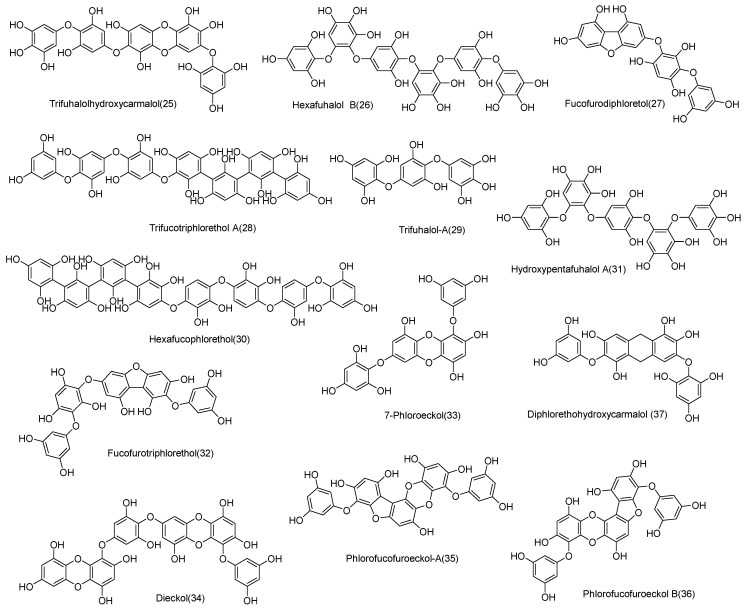
Chemical structures of representative seaweed-derived polyphenolic compounds discussed in this review. Compounds **25**–**37** are a continuation of the polyphenolic compounds presented in Figure 2.

The structure–activity relationship (SAR) of seaweed polyphenols is a key determinant underlying their anti-hyperglycemic activity. Phlorotannins with higher molecular weight and greater hydroxyl group density generally exhibit stronger inhibitory effects on α-glucosidase and α-amylase. Selective inhibition of α-glucosidase over α-amylase—observed in extracts from species such as *Durvillaea antarctica* and *Ascophyllum nodosum*—suggests that specific structural features, including degree of polymerization and substitution patterns, confer enzyme selectivity [22,26]. Interactions between polyphenolic functional groups and enzyme-active or allosteric sites involve hydrogen bonding and hydrophobic interactions, strengthened by the presence of multiple hydroxyl groups and conjugated aromatic rings. The chemical structures of the representative seaweed-derived polyphenolic compounds discussed in this section are illustrated in Figure 2 and Figure 3.

Extraction techniques also play a crucial role in shaping SAR-related outcomes. Methods such as ethanol-based extraction, pressurized hot water extraction, and ultrasound-assisted extraction have been shown to enhance the yield of bioactive polyphenols and preserve functional group integrity [27,28]. Recent optimization studies on phlorotannin extraction from Arctic *Fucus vesiculosus* further demonstrated that solvent selection and extraction parameters markedly influence phlorotannin yield, chemical profiling, and radical-scavenging activity. In particular, natural deep eutectic solvents combined with ultrasound-assisted extraction provided a high phlorotannin yield and enabled the identification of 32 phlorotannins, while maintaining antioxidant activity comparable to that of ethanol extracts. These findings indicate that the extraction solvent is not only a technical variable for recovery efficiency, but also a determinant of the compositional profile and bioactivity of seaweed polyphenols [29]. Furthermore, non-extractable polyphenols from *Undaria pinnatifida*, enriched in ferulic acid and p-coumaric acid derivatives, have been shown to improve glucose metabolism and insulin resistance by modulating the IRS–PI3K–Akt–GLUT4 signaling pathway [19]. In summary, the type, number, and position of functional groups such as hydroxyl, carboxyl, and quinone moieties collectively determine the glucose-regulating effects of seaweed polyphenols [27,30].

## 3. Seaweed-Derived Polysaccharides and Diabetes

### 3.1. Overview of Seaweed Polysaccharides

#### 3.1.1. Sources and Classification of Seaweed Polysaccharides

Seaweed polysaccharides are a class of natural macromolecules primarily derived from the cell walls and extracellular matrices of marine macroalgae, exhibiting remarkable structural diversity [4]. In contrast to terrestrial plant polysaccharides—which are typically composed of glucose as the predominant monosaccharide and mainly serve structural and energy storage functions—seaweed polysaccharides display greater structural complexity and heterogeneity. Marine algae are continuously exposed to high salinity, elevated ionic strength, and dynamic aquatic environments, which have driven the evolution of more complex and functionally specialized polysaccharide structures.

According to algal taxonomy, seaweed polysaccharides are generally classified into three major categories: brown algal polysaccharides (predominantly alginate, fucoidan, and laminarin), red algal polysaccharides (rich in sulfated galactans such as agar and carrageenan), and green algal polysaccharides (characterized by ulvan, a sulfated heteropolysaccharide mainly composed of rhamnose and uronic acids) [4]. These polysaccharides differ significantly in terms of monosaccharide composition, glycosidic linkage patterns, and degree of sulfation, which collectively determine their physicochemical properties and biological functions.

The unique structural characteristics of seaweed polysaccharides—particularly their diverse monosaccharide composition and the presence of sulfated groups—are closely associated with their wide range of bioactivities. These include antioxidant, anti-inflammatory, immunomodulatory, and glucose-regulating effects [7]. These multi-target biological effects highlight the significant potential of seaweed polysaccharides in the prevention and management of metabolic disorders, particularly T2DM.

#### 3.1.2. Structural Characteristics of Seaweed Polysaccharides

**Figure 4:**
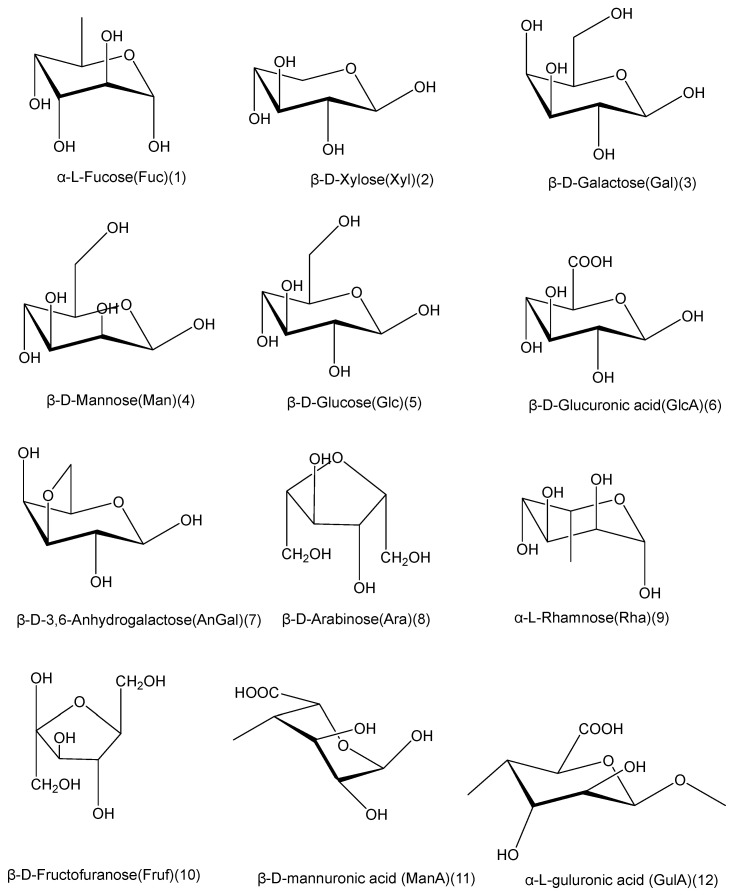
Chemical structures of representative monosaccharide constituents of seaweed-derived polysaccharides.The composition, relative abundance, and glycosidic-linkage patterns of these monosaccharides contribute to the structural diversity and biological properties of seaweed polysaccharides.

One of the most distinctive structural features of seaweed polysaccharides is their highly diverse monosaccharide composition, which includes fucose, xylose, mannose, galactose, glucose, and glucuronic acid [31]. For example, fucoidan is primarily composed of sulfated α-L-fucose residues, whereas alginate consists of β-D-mannuronic acid and α-L-guluronic acid units arranged in varying block structures. In contrast, ulvan derived from green algae is characterized by a high content of rhamnose and uronic acids. The representative monosaccharide constituents of seaweed-derived polysaccharides discussed herein are illustrated in Figure 4.

**Figure 5:**
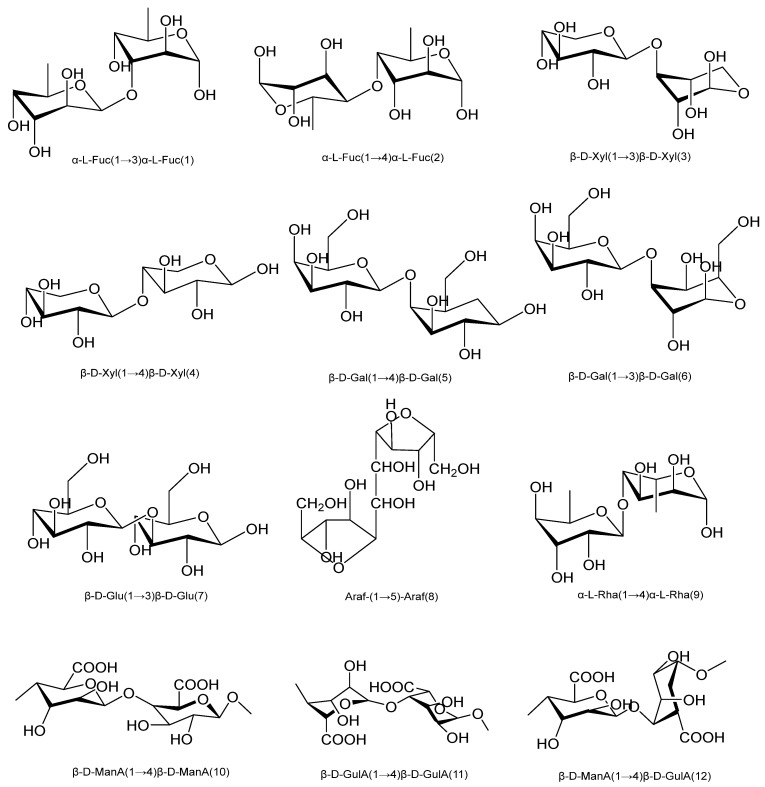
Representative glycosidic linkage patterns occurring in seaweed-derived polysaccharides. These linkage patterns contribute to the structural diversity and physicochemical and biological properties of seaweed polysaccharides.

Glycosidic linkage patterns further contribute to structural complexity. Fucoidan can be classified into three main types based on its glycosidic linkages: (1→3)-linked α-L-fucopyranose chains, (1→4)-linked structures, or alternating (1→3)/(1→4)-linked configurations [32]. This variability directly influences the degree and distribution of sulfation, as well as branching patterns, thereby modulating biological activities [33]. Alginate is a linear copolymer composed of β-D-mannuronic acid (M) and α-L-guluronic acid (G) residues linked via (1→4) glycosidic bonds, with the relative proportion and sequential arrangement of M and G blocks critically determining gel-forming properties [32]. The representative glycosidic linkage patterns between monosaccharide residues in seaweed-derived polysaccharides are illustrated in Figure 5.

Sulfation represents one of the most distinctive structural features differentiating seaweed polysaccharides from those of terrestrial plants. Both the degree of sulfation and the substitution pattern play critical roles in modulating functional properties [34]. Sulfate groups confer a pronounced negative charge, facilitating electrostatic interactions with proteins and enzymes while also influencing molecular conformation and binding affinity [35]. These structural characteristics significantly enhance hydration capacity, water solubility, and overall biological activity.

### 3.2. Glucose-Regulating Mechanisms of Seaweed Polysaccharides

Seaweed-derived polysaccharides exert their effects through multi-target and multi-level regulatory mechanisms, thereby systemically ameliorating key pathological processes of T2DM. Current evidence indicates that their glucose-regulating and metabolism-modulating effects mainly involve attenuation of chronic low-grade inflammation and oxidative stress, regulation of glucose and lipid metabolic homeostasis, improvement of insulin resistance, and modulation of gut microbiota composition, reflecting a characteristic ‘holistic intervention’ mode of action (Table 2).

#### 3.2.1. Anti-Inflammatory Mechanisms

Polysaccharides derived from marine algae have demonstrated significant anti-inflammatory properties, which are critical for alleviating chronic inflammation and insulin resistance in T2DM. A key mechanism involves the suppression of pro-inflammatory cytokines such as TNF-α, IL-6, and IL-1β [36]. For example, polysaccharides from *Undaria pinnatifida* and *Fucus vesiculosus* effectively inhibit the production of these cytokines in human peripheral blood mononuclear cells (PBMCs) [37]. At the molecular level, sulfated polysaccharides such as fucoidan directly scavenge reactive oxygen species (ROS), which leads to decreased expression of inflammatory mediators [38,39].

The gut microbiota also plays a crucial role in mediating the anti-inflammatory effects of polysaccharides. Many polysaccharides act as prebiotics, modulating gut microbial composition and promoting the production of short-chain fatty acids (SCFAs). These metabolites activate signaling pathways such as AMPK and IRS-1 in the liver, reduce pro-inflammatory cytokines, and increase anti-inflammatory cytokines such as IL-10 [40]. Furthermore, polysaccharides enhance endogenous antioxidant defense systems by upregulating enzymes such as SOD and CAT, creating a favorable microenvironment for restoring insulin sensitivity [41].

#### 3.2.2. Antioxidative Stress Mechanisms

Seaweed polysaccharides exhibit potent antioxidant activities that are directly relevant to metabolic homeostasis in T2DM. Their abundant sulfate and carboxyl groups enable direct scavenging of reactive oxygen species (ROS), thereby reducing oxidative stress-induced damage to pancreatic β-cells and peripheral tissues [42]. For example, sulfated polysaccharides derived from *Porphyra haitanensis* exhibit greater resistance to α-amylase-mediated digestion compared to non-sulfated carbohydrates, suggesting that higher sulfate levels confer enhanced structural stability and potential prebiotic effects [43]. In addition to sulfate and carboxyl groups, associated polyphenols and monosaccharide composition may also shape the antioxidant and anti-inflammatory activities of fucoidans. A comparative study of fucoidans from five brown seaweed species showed that total antioxidant capacity was strongly and positively correlated with polyphenol content, while xylose content showed only a weak association. Moreover, synergistic effects between carbohydrate and polyphenol components were observed in the DPPH assay for two *Fucus vesiculosus* fucoidans, FV1 and FV3, with mixture effect values of 2.68 and 2.04, respectively. These findings suggest that the bioactivity of fucoidans should be interpreted as the result of both polysaccharide structural features and associated phenolic components, rather than sulfate content alone [52].

By reducing intracellular ROS levels and alleviating lipid peroxidation (decreased MDA), and by upregulating endogenous antioxidant enzymes (SOD, CAT, GPx), seaweed polysaccharides protect β-cells from oxidative damage, preserve insulin secretion capacity, and ameliorate oxidative stress-induced insulin resistance [44]. These antioxidant effects synergize with their anti-inflammatory properties to comprehensively address the oxidative-inflammatory axis central to T2DM pathogenesis [53,54].

#### 3.2.3. Enhancement of Insulin Secretion and Insulin Sensitivity

Seaweed-derived polysaccharides have demonstrated significant potential in modulating insulin secretion and sensitivity to restore glucose homeostasis. The polysaccharide fraction SFP-2 isolated from *Sargassum fusiforme* significantly improves insulin sensitivity as evidenced by reductions in HOMA-IR indices [45]. Polysaccharides from *Fucus vesiculosus* inhibit dipeptidyl peptidase-IV (DPP-IV), thereby prolonging the activity of endogenous incretin hormones and enhancing insulin secretion [46,47]. Seaweed polysaccharides from *Gracilaria gracilis* activate the IRS-1-mediated insulin signaling cascade and promote the expression and translocation of glucose transporter 4 (GLUT4), thereby enhancing peripheral glucose uptake [48].

The polysaccharide fraction UP-4 isolated from *Undaria pinnatifida* not only regulates postprandial glucose levels by inhibiting α-glucosidase activity, but also enhances glucose uptake in HepG2 hepatocytes, indicating a multi-target mechanism for improving glycemic control. These findings collectively highlight the therapeutic potential of seaweed polysaccharides as natural compounds for improving insulin dynamics and reducing hyperglycemia in T2DM [55].

#### 3.2.4. Inhibition of Carbohydrate-Hydrolyzing Enzymes

Inhibition of α-amylase and α-glucosidase represents an important therapeutic strategy for managing T2DM by controlling postprandial hyperglycemia. Seaweed polysaccharides have demonstrated promising potential as inhibitors of these digestive enzymes [49,50]. Notably, the inhibitory activities of seaweed polysaccharides against these enzymes are closely associated with their degree of sulfation, with higher sulfation levels generally correlating with enhanced anti-hyperglycemic efficacy.

Polysaccharides from *Ecklonia maxima* exhibit strong inhibitory activity against α-glucosidase while exerting minimal effects on α-amylase, thereby achieving glycemic control without inducing significant gastrointestinal side effects [51]. The multi-target nature of these marine bioactive compounds enables them to modulate multiple pathways involved in T2DM pathogenesis. Future research should further explore the chemical diversity of marine-derived compounds and elucidate the precise mechanisms underlying their enzyme-inhibitory activities [56].

#### 3.2.5. Other Anti-Hyperglycemic Mechanisms

Beyond the well-characterized mechanisms, seaweed-derived polysaccharides exhibit additional bioactivities contributing to the potential for diabetes management. One emerging mechanism involves modulation of the gut microbiota. Seaweed polysaccharides, including fucoidan, laminarin, alginate, ulvan, and porphyran, function as prebiotics, selectively promoting the growth of beneficial gut bacteria. This modulation enhances the production of SCFAs, improves gut barrier integrity, and exerts systemic anti-inflammatory effects, thereby contributing to T2DM management [57].

Marine-derived peptides and polyphenols have also been reported to exert multi-target regulatory effects on glucose and lipid metabolism, influencing key signaling pathways associated with insulin sensitivity, glucose uptake, and lipid homeostasis [56]. Other seaweed bioactive constituents, including unsaturated fatty acids and dietary fibers, contribute to metabolic regulation by delaying gastric emptying and enhancing insulin signaling. The unique structural features and compositional diversity of these marine bioactives enable interactions with multiple metabolic targets, providing a promising resource for novel agents with potential for diabetes management [58].

### 3.3. Structure–Activity Relationships of Seaweed Polysaccharides

The biological activities of seaweed polysaccharides are fundamentally determined by their structural characteristics. Among these, the degree and distribution pattern of sulfation, molecular weight, and the extent of methylation and acetylation are recognized as key factors regulating their physicochemical properties and biological functions. These structural features interact synergistically to collectively influence the overall bioactivity.

#### 3.3.1. Sulfation

The sulfate content of marine-derived polysaccharides is increasingly recognized as a critical determinant of their biological activity, particularly in their inhibitory effects on α-amylase and α-glucosidase. Seaweed-derived polysaccharides exhibit varying degrees of inhibitory activity toward these enzymes, closely associated with variations in sulfate content. Sulfated polysaccharides derived from *Porphyra haitanensis* exhibit greater resistance to α-amylase-mediated digestion, suggesting that higher sulfate levels confer enhanced structural stability [43]. Sulfate groups facilitate specific interactions with enzyme-active sites, induce conformational changes, and influence enzyme kinetics.

#### 3.3.2. Molecular Weight

The molecular weight (Mw) of seaweed-derived polysaccharides represents a critical structural determinant that governs biological activities including pharmacokinetics, bioavailability, and therapeutic efficacy. High-molecular-weight fucoidan (HMWF) is often limited by poor water solubility, high viscosity, and restricted bioavailability. To overcome these limitations, low-molecular-weight fucoidan (LMWF) and fucoidan-derived oligosaccharides have been extensively investigated, displaying improved physicochemical properties while retaining or surpassing the bioactivities of their high-Mw counterparts. LMWF hydrolysates (<1.5 kDa) significantly reduce pro-inflammatory cytokines TNF-α, IL-1β, and IL-6, highlighting their enhanced anti-inflammatory potential [59].

Low-Mw acidic polysaccharides have demonstrated superior α-glucosidase inhibitory activity in vitro and significantly reduced fasting blood glucose and glycated hemoglobin (HbA1c) levels in diabetic KK-Ay mice, suggesting a strong correlation between reduced Mw and enhanced anti-hyperglycemic activity [60]. Similarly, enzymatically generated low-Mw ulvan oligosaccharides exhibit improved solubility, biocompatibility, and enhanced antioxidant and immunomodulatory activities relative to their native high-Mw forms [61]. Mw optimization may therefore serve as an effective strategy to tailor polysaccharide bioactivity for specific therapeutic applications.

Pharmacokinetic behavior and tissue distribution are also important for interpreting the in vivo activity of fucoidan. Recent research reported that orally administered fucoidan from *Fucus vesiculosus* could be detected in rat plasma and tissues, with heterogeneous tissue distribution and preferential accumulation in organs with strong filtering functions, particularly the kidney, spleen, and liver. These findings suggest that the biological effects of fucoidan are influenced not only by molecular weight, sulfation level, and monosaccharide composition, but also by absorption, systemic exposure, and organ-specific distribution [62].

#### 3.3.3. Methylation and Acetylation

The anti-hyperglycemic activity of marine polysaccharides is not solely determined by sulfation but is also significantly influenced by methylation and acetylation. Methylation can enhance the hydrophobicity of polysaccharides, potentially improving their cellular uptake and facilitating interactions with key enzymes involved in glucose metabolism. Acetylation can alter the charge distribution and steric configuration of polysaccharides, affecting their ability to interact with receptors and enzymes associated with insulin sensitivity and glycemic control.

Both modifications may also modulate the antioxidant properties of polysaccharides, indirectly alleviating insulin resistance and β-cell dysfunction [58]. A deeper understanding and precise control of these modifications will be essential for maximizing hypoglycemic efficacy and facilitating the development of effective marine-derived agents with potential for diabetes management [56].

#### 3.3.4. Extraction Techniques

For polysaccharides, extraction procedures exert an even stronger influence on molecular weight, sulfate content, and monosaccharide composition. In Ulva clathrata, hot-water, alkaline, acid, enzymatic, ultrasound-assisted, and microwave-assisted extraction produced polysaccharide yields of 21.5%, 23.4%, 26.3%, 22.6%, 15.6%, and 17.3%. Although acid extraction gave the highest yield, it markedly reduced the average molecular weight to 185.1 kDa, compared with 928.5 kDa for hot-water extraction, indicating extensive chain cleavage [63].

Recent work on fucoidan further supports the importance of solvent selection. Replacing conventional alcohol precipitation with citric acid extraction combined with molecular-weight-cut-off filtration enriched high-purity fucoidan, with the >300 kDa fractions containing 79.16% fucoidan from *Fucus vesiculosus* and 79.21% from *Ascophyllum nodosum* and showing stronger antioxidant activity than crude extracts [64]. Therefore, the extraction strategy should not be regarded merely as a preparative step, but as a key factor governing the structure–activity relationship and translational value of seaweed-derived polysaccharides.

## 4. Discussion

This review systematically summarizes the structural characteristics, biological activities, and glucose-regulating mechanisms of seaweed-derived polyphenols and polysaccharides, highlighting their potential as multifunctional natural agents for the management of T2DM. A key insight emerging from the current body of evidence is that both classes of compounds exhibit multi-target and multi-level regulatory effects, which align well with the complex and multifactorial pathophysiology of T2DM.

One of the central themes identified in this review is the critical role of structural features in determining bioactivity. The structural features of representative active compounds derived from seaweeds are summarized in Table 3. For seaweed polyphenols, particularly phlorotannins, parameters such as the degree of polymerization, hydroxyl group density, and linkage patterns directly influence antioxidant capacity, enzyme inhibition, and signaling pathway modulation. Similarly, for polysaccharides, the structural determinants such as their degree of sulfation, molecular weight, monosaccharide composition, and the connection mode of glycosidic bonds jointly determine their physical and chemical properties, as well as their biological functions. Notably, sulfation emerges as a key functional modification that enhances electrostatic interactions with proteins and enzymes, thereby improving activities such as α-glucosidase inhibition and anti-inflammatory signaling. Importantly, the SAR of these marine-derived compounds is not governed by a single factor but rather by the synergistic interplay of multiple structural parameters. For instance, while high-molecular-weight polysaccharides may exhibit strong bioactivity, their poor bioavailability limits clinical translation; conversely, low-molecular-weight derivatives often demonstrate improved absorption and enhanced biological effects. This highlights the necessity of optimizing structural features through controlled extraction, modification, or depolymerization strategies.

**Figure 6:**
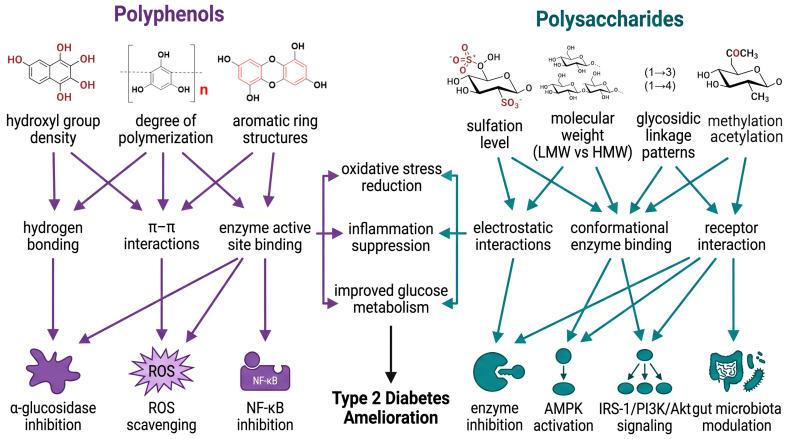
The structure–activity relationship of polysaccharides and polyphenols underlying their glucose-regulating effects (Purple: Algal polyphenols; Blue color: Algal polysaccharide; Red: The structural features that are of concern; Dotted line: Groups that undergo derivatization).

Mechanistically, both polyphenols and polysaccharides converge on several core pathological processes of T2DM, including oxidative stress, chronic inflammation, insulin resistance, and dysregulated glucose and lipid metabolism. Polyphenols primarily exert strong antioxidant and enzyme-inhibitory effects, directly scavenging reactive oxygen species and suppressing carbohydrate digestion. In contrast, polysaccharides exhibit broader systemic regulation, including gut microbiota modulation, immune regulation, and enhancement of intestinal barrier function. This distinction suggests a potential complementary or synergistic effect between these two classes of compounds, which may be advantageous for integrated therapeutic strategies. In addition to the multi-target characteristics of individual compounds mentioned above, we have proposed a viewpoint that the polyphenols and polysaccharides derived from seaweed exert their effects in a spatial and temporal integrated coordinated manner to combat T2DM. This viewpoint suggests that the therapeutic effect of the entire seaweed extract or combined formulation may exceed the sum of its individual components, because its components have sequential and compartmentalized effects. This potential integrated synergy mainly functions at two levels. Firstly, it affects the interaction between cavities and tissues: high-molecular-weight polysaccharides and non-absorbable anthocyanin polyphenols mainly act in the gastrointestinal cavity. Here, polysaccharides inhibit α-amylase and delay the initial breakdown of complex carbohydrates, while polyphenols strongly inhibit α-glucosidase on the brush border and directly inhibit the spike in post-meal blood sugar [10]. Secondly, it maintains the transmission mechanism between the intestine and organs: these two compound categories jointly weaken oxidative stress in the intestine and the inflammatory effects triggered by NF-κB [38], which can maintain the integrity of the intestinal barrier and reduce the leakage of inflammatory triggers. This intracellular anti-inflammatory effect creates a favorable systemic environment. At the same time, short-chain fatty acids and small amounts of absorbed low-molecular-weight phenolic substances directly activate AMPK in the liver and upregulate the IRS-1/PI3K/Akt signaling pathway in muscle and fat tissues, thereby systematically improving insulin sensitivity [4]. This integrated coordination mechanism explains why the total extract sometimes outperforms the purified isolated substances and provides a strong pharmacological basis for exploring multi-component rather than single-compound treatment strategies to achieve overall management of T2DM.The key structural determinants and associated glucose-regulating mechanisms of seaweed-derived polyphenols and polysaccharides are summarized in Figure 6.

Another notable aspect is the emerging role of the gut–metabolism axis. Seaweed polysaccharides function as prebiotics, promoting the production of SCFAs and regulating host metabolic signaling pathways such as AMPK and IRS-1. This indirect mechanism represents a paradigm shift from traditional glucose-lowering strategies toward host–microbiota co-regulation, offering new opportunities for intervention in metabolic diseases.

Despite these promising findings, several challenges remain. First, most current evidence is derived from in vitro and preclinical studies, with limited well-controlled clinical trials to validate efficacy and safety in humans. Second, the structural heterogeneity and lack of standardization in seaweed-derived compounds hinder reproducibility and comparability across studies. Third, issues related to bioavailability, metabolic stability, and pharmacokinetics remain insufficiently addressed, particularly for high-molecular-weight or highly polymerized compounds such as phlorotannins. Furthermore, while SAR studies have provided valuable insights, they are often fragmented and lack integration with advanced computational or systems-level approaches. The application of modern technologies, such as metabolomics, network pharmacology, artificial intelligence-driven modeling, and molecular docking, will be essential to establish more precise structure–function relationships and identify key bioactive motifs.

Overall, this review underscores the importance of integrating chemical structure, biological function, and mechanistic pathways to fully understand and exploit the therapeutic potential of seaweed-derived bioactive compounds. Future research should prioritize standardization, mechanistic depth, and clinical translation, as well as explore combinatorial strategies involving polyphenols and polysaccharides.

## 5. Conclusions

Seaweed-derived polyphenols and polysaccharides represent a diverse and promising class of marine natural products with significant potential for the prevention and management of type 2 diabetes mellitus. Their unique structural features underpin a wide range of biological activities, including antioxidant, anti-inflammatory, enzyme-inhibitory, and metabolic-regulatory effects. A major advantage of these compounds lies in their multi-target mechanisms of action, enabling simultaneous modulation of key pathological processes in T2DM, including oxidative stress, insulin resistance, lipid dysregulation, and gut microbiota imbalance. The strong correlation between structural characteristics and bioactivity further highlights the importance of structure–activity relationship studies in guiding the development of effective therapeutic agents. However, the clinical application of seaweed-derived compounds is still limited by challenges such as structural heterogeneity, low bioavailability, and insufficient clinical validation. Addressing these limitations will require advances in extraction technologies, structural characterization, delivery systems, and translational research. In the future, the integration of chemical modification, nanotechnology-based delivery, and systems biology approaches is expected to enhance the efficacy and applicability of these marine bioactives. Moreover, the development of functional foods, nutraceuticals, and combination therapies based on seaweed-derived compounds holds considerable promise for improving metabolic health. In conclusion, seaweed-derived polyphenols and polysaccharides provide a valuable resource for the development of novel, safe, and effective strategies for diabetes management. Continued interdisciplinary research will be essential to unlock their full therapeutic potential and facilitate their transition from experimental studies to clinical applications.

## Figures and Tables

**Table 1:** Marine-derived polyphenols showing glucose-regulating effects and related mechanisms of action.

Seaweed Source	Polyphenolic Compound	Extract Type	Study Type and Model	Dose/Concentration Range	Minimal Active Concentration	Controls (Positive/Negative)	Duration	Bioactivity and Mechanism	Reference
*Ecklonia stolonifera*	(**34**)(**35**)	Purified phlorotannins	In vitro: Cell-based GPCR functional screening assays; in silico molecular docking analysis	1–100 µM (cell-based assays);	~1 µM	Positive control: reference agonists/antagonists for each receptor; Negative control: DMSO	Single-dose assay (no prolonged treatment)	GLP-1 receptor signaling modulation↓, suggesting a potential role in the modulation of glucose homeostasis	[13]
*Ulva fasciata*	(**1**)(**2**)(**3**)(**4**)(**5**)(**6**)(**7**)(**8**)(**9**)(**10**)(**11**)(**12**)(**13**)(**16**)	Methanolic extract	In vivo: L-thyroxine-induced hyperthyroid rat model	In vivo: 200 mg/kg b.w. (oral); L-thyroxine: 100 µg/kg i.p. (3 weeks, daily); Propranolol HCl: 10 mg/kg i.p.	200 mg/kg b.w.	Positive control: propranolol hydrochloride (10 mg/kg i.p.); Negative control: normal rats	3 weeks (daily treatment)	ROS↓ and inhibiting lipid peroxidation	[15]
*Eisenia bicyclis*	(**34**)	Ultrasound-assisted ethanol extract	In vitro: ABTS•^+^ radical scavenging assay	Dieckol extract at 0–1000 µg/mL	_____	Positive control: Trolox or ascorbic acid; Negative control: ABTS•^+^ reagent without extract	Single-dose in vitro assay	Exhibits significant antioxidant activity by donating electrons and hydrogen atoms to scavenge free radicals	[16]
*Saccharina japonica*	(**17**)(**18**)(**19**)(**20**)(**21**)(**22**)(**23**)(**24**)(**25**)(**26**)(**27**)(**28**)(**29**)(**30**)(**31**)(**32**)	Ethanol extract	In vitro: DPPH• and ABTS•^+^ scavenging assays; HepG2 cell oxidative damage model	Chemical assays: 0.1–5 mg/mL; HepG2 cell model: extract at various concentrations (0.1–1 mg/mL)	Concentration-dependent; high-MW fractions (>50 kDa) show higher activity than low-MW fractions	Positive control: Trolox; Negative control: DMSO	Single-dose in vitro assay	ROS↓, MDA↓, SOD↑, thereby attenuating oxidative stress-induced cellular damage	[17]
*Ecklonia cava*	(**33**)	Ethanol extract	In vitro: HepG2/CYP2E1 cells	0, 12.5, 25, 50, 100 µM (dose-response); 50 µM as the primary test concentration	12.5 µM	Positive control: N-acetyl-L-cysteine (NAC); Negative control: DMSO; Model control: 600 mM ethanol-treated cells	24 h treatment	ROS↓, NO↓, NF-κB↓, JNK↓, thereby attenuating oxidative stress; Bax/Bcl-2↓, caspase-3↓, alleviating ethanol-induced hepatocellular injury	[18]
*Undaria pinnatifida*	(**6**)(**7**)(**8**)(**12**)(**13**)(**14**)(**15**)	Non-extractable polyphenols (NEPPs) via alkaline hydrolysis with ultrasonic assistance	In vitro: α-amylase inhibition assay; DPPH•, ABTS•^+^, OH• scavenging assays; DPP-IV inhibition assay In vivo: HFD/STZ-induced T2D mice	In vitro: 0.1–5 mg/mL (enzyme inhibition); In vivo: 400 mg/kg/day; Metformin: 200 mg/kg/day	In vitro: IC_50_ for α-glucosidase inhibition: 0.69 mg/mL; In vivo: effective at 400 mg/kg/day	Positive controls: acarbose; metformin 200 mg/kg (in vivo); Negative control: model group (HFD/STZ, vehicle-treated)	In vivo: 8 weeks of HFD + STZ induction; treatment for additional 4 weeks	Carbohydrate-hydrolyzing enzyme↓, thereby modulating glucose absorption; oxidative stress↓, which is closely linked to insulin resistance	[19]
*Ecklonia stolonifera*	(**36**)	Ethanolic extract, purified by HPLC	In vitro: LPS-stimulated BV-2 microglial cells	PFF-B: 1, 5, 10, 20, 40 µM; primary effective range: 10–40 µM	10 µM	Positive control: LPS (1 µg/mL); Negative control: untreated BV-2 cells; Solvent control: DMSO	24–48 h treatment (standard for LPS-stimulated in vitro assay)	NO↓, PGE_2_↓, Cox-2↓, iNOS↓; Akt, ERK, and JNK signaling pathways↓ demonstrate potential anti-inflammatory effects	[20]
*Ishige okamurae*	(**37**)	Ethanolic extract	In vitro:3T3-L1 preadipocytes and differentiated adipocytes (Oil Red O staining for lipid accumulation)	DPHC: 1, 5, 10, 25, 50 µM; differentiation induced by standard adipogenic cocktail	10 µM (significant inhibition of lipid accumulation and adipogenic proteins)	Positive control: differentiated adipocytes (MDI-induced); Negative control: undifferentiated 3T3-L1 preadipocytes; Solvent control: DMSO	8 days of differentiation (with DPHC treatment)	AMPK↑, SREBP-1c↓, PPARγ↓, C/EBPα↓, FAS↓; inhibits lipid accumulation and demonstrates potential glucose-regulating effects	[21]
*Ascophyllum nodosum*	(**33**)	Acetone extract and HPLE (hot pressurized liquid ethanol/water extract)	In vitro: α-amylase and α-glucosidase inhibition assay	0–1000 µg/mL	IC_50_ for α-glucosidase: approximately comparable to acarbose at ≥100 µg/mL; α-amylase inhibition moderate	Positive control: acarbose; Negative control: enzyme + substrate without extract	Single-dose in vitro assay	Carbohydrate-hydrolyzing enzyme↓, leading to postprandial glucose release↓ and delayed starch hydrolysis	[22]

Note: ↑ and ↓ indicate increased or decreased levels, expression, activity, or signaling, respectively, compared with the corresponding control group.

**Table 2:** Marine-derived polysaccharides showing glucose-regulating effects in pre-clinical studies and related mechanisms of action.

Seaweed Source	Monosaccharide Composition	Glycosidic Linkage	Extract/Polysaccharide Type	Study Type and Model	Dose/Concentration Range	Minimal Active Concentration	Controls (Positive/Negative)	Duration	Bioactivity and Mechanism	Reference
*Gelidium pacificum Okamura*	Gal, Xyl, GalA;	→4)-α-D-Galp3S-(1→2)-α-D-Xylp-(1→3)-β-D-GalpA-(1→	Sulfated polysaccharide (GPOP-1) extracted by hot water + ultrasound at 80 °C	In vitro: LPS-stimulated human monocytic THP-1 cells	1.25, 2.5, 5 µg/mL (dose–response)	5 µg/mL	Positive control (inflammation): LPS (2 µg/mL) alone; Negative control: untreated THP-1 cells; Treatment groups: GPOP-1 pre-treatment (1 h) + LPS co-treatment	24 h treatment	TLR4/MyD88/TRAF6 signaling↓, leading to NO↓	[36]
*Undaria pinnatifida*	Fuc, Gal	α-L-Fuc-(1→3)-α-L-Fuc	Fucoidan extracts (commercial fucoidan);	In vitro: RAW264.7 macrophages, THP-1 cells, PBMCs (LPS-stimulated), Caco-2 cells; LPS: 1 µg/mL as stimulant	1, 10, 100, 1000 µg/mL	10 µg/mL	Positive control: LPS (1 µg/mL) stimulation; Negative control: untreated cells; Internal comparison: multiple fucoidan sources	24–48 h treatment	TNF-α↓, IL-1β↓, IL-6↓	[37]
*Undaria pinnatifida*	Fuc	→3)-α-L-Fucp-(1→4)-α-L-Fucp-(1→	Fucoidan extract from U. pinnatifida (commercial)	In vitro: ARPE-19 retinal pigment epithelial cells infected with HSV-1 (herpes simplex virus type 1)	5, 10, 50, 100, 200 µg/mL	50 µg/mL	Positive control: virus-infected ARPE-19 cells without fucoidan; Negative control: uninfected cells; Antioxidant reference: Trolox	24–72 h	ROS↓; suppression of NF-κB p65 phosphorylation and nuclear translocation; IL-6↓; indirect attenuation of inflammation-associated amyloid-β synthesis	[38]
*Laminaria japonica*	Fuc, Gal, GlcA	→3)-α-L-Fucp-(1→4)-α-L-Fucp-(1→	Fucoidan hydrolysates: intact fucoidan (F);	In vitro: LPS-stimulated Caco-2/RAW264.7 co-culture model (LPS: 1 µg/mL)	Fucoidan, LMAF, HMAF at 25, 50, 100, 200 µg/mL	LMAF at 50 µg/mL	Positive control: LPS (1 µg/mL) stimulation; Negative control: untreated cells; Comparison: intact fucoidan vs. LMAF vs. HMAF	24 h LPS stimulation + concurrent treatment	Modulates the TNF and NF-κB signaling pathways, thus NO↓, TNF-α↓, IL-1β↓, and IL-6↓	[39]
*Laminaria japonica*	Fuc	→4)-α-L-Fucp-(1→3)-α-L-Fucp-(1→	Extracted by hot water; purified by DEAE-cellulose and Sephadex G-100	In vivo: HFD- and streptozotocin (STZ)-induced T2DM mice	50, 100, 200 mg/kg/day; STZ: 35 mg/kg i.p. (single dose after 4 weeks HFD); Metformin: 200 mg/kg/day (positive drug)	100 mg/kg/day	Positive control (drug): metformin (200 mg/kg/day); Negative control: HFD/STZ model group (vehicle); Normal control: standard diet + vehicle	4 weeks HFD + STZ induction; 8 weeks polysaccharide treatment	SCFAs↑, activation of hepatic AMPK/IRS-1 signaling, IL-6↓	[40]
*Undaria pinnatifida*	Fuc	→3)-α-L-Fucp-(1→4)-α-L-Fucp-(1→	*Undaria pinnatifida* fucoidan (UPF; commercial high-purity fucoidan)	In vivo: Salmonella typhimurium-infected mouse model	UPF: 50, 100, 200 mg/kg/day; Antibiotic (streptomycin 20 mg/mouse, 1 day before infection)	100 mg/kg/day	Positive control: antibiotic-treated infected mice; Negative control: infected vehicle-treated mice; Normal control: uninfected mice	Pre-treatment: 7 days; post-infection monitoring: 14 days	SOD↑, CAT↑, MDA↓, iNOS↓, ROS accumulation↓; NF-κB signaling pathway↓; decreased production of pro-inflammatory cytokines and alleviation of intestinal inflammation	[41]
*Gloiopeltis furcata*	Gal, AnGal	→3)-β-D-Gal-(1→ and →4)-α-L-AnGal-(1→	Extracted by hot water + enzymatic hydrolysis;	In vitro: PA-induced insulin-resistant HepG2 cells (sodium palmitate 0.25 mM for 24 h induction)	SAOs: 0.1, 0.5, 1.0, 2.0 mg/mL (dose–response)	0.5 mg/mL	Positive control: insulin (100 nM); Negative control: PA-treated cells without SAOs; Normal control: untreated HepG2 cells	24–48 h treatment	ROS↓; ROS/JNK/c-Jun signaling pathway↓, intracellular oxidative stress↓	[42]
*Porphyra haitanensis*	Gal, Glc, Fuc;	→3)β-D-Gal(1→ and →4)β-D-Gal(1→	Extracted by water extraction and alcohol	In vitro: DPPH•, ABTS•^+^, and OH• radical scavenging assays	1–5 mg/mL	IC_50_ for ABTS•^+^ scavenging: ~1 mg/mL	Positive control: Vitamin C or Trolox; Negative control: buffer without polysaccharide	Single-dose in vitro assay	Exhibits concentration-dependent scavenging of ROS, including DPPH•, ABTS•^+^, and OH•, thereby alleviating oxidative stress	[43]
*Low-molecular-weight fucoidan (LMWF) (brown algae-derived)*	Fuc	→3)-α-L-Fucp-(1→4)-α-L-Fucp-(1→	Low-molecular-weight fucoidan (LMWF; commercial; MW <10 kDa) derived from brown algae	In vivo: Aged mouse model of traumatic brain injury (TBI): subjected to controlled cortical impact (CCI) model	LMWF: 1, 10, 50 mg/kg i.p. (post-injury administration);	10 mg/kg	Positive control: vehicle-treated TBI mice; Negative control: sham-operated mice; Sirt3 knockdown group: siRNA injection; Comparison: young vs. aged TBI	Up to 28 days post-TBI (neurological function assessment)	Sirt3↑, ROS↓, CAT↑, SOD↑, and GPX↑	[44]
*Sargassum fusiforme*	Fuc, Gal, Man, GlcA	____	Extracted by ultrasound-assisted enzymatic extraction from *S. fusiforme*	In vivo: HFD- and streptozotocin (STZ)-induced T2DM rats	SFP-1 and SFP-2: 200, 400, 800 mg/kg/day; STZ: 35 mg/kg i.v. (after 4 weeks HFD); Metformin: 200 mg/kg/day	SFP-2 at 400 mg/kg/day	Positive control (drug): metformin (200 mg/kg/day); Negative control: T2DM model group (vehicle); Normal control: standard diet + vehicle	4 weeks HFD + STZ induction; 8 weeks treatment	HOMA-IR↓ (insulin sensitivity improved); lipid profiles improved; liver and kidney function protected	[45]
*Fucus vesiculosus*	Fuc, GlcA	____	____	In vivo: HFD- and streptozotocin (STZ)-induced T2DM rats	FVP: 400 mg/kg/day; STZ: 35 mg/kg i.v. (after 4 weeks HFD); Metformin: 200 mg/kg/day	400 mg/kg/day	Positive control (drug): metformin (200 mg/kg/day); Negative control: T2DM model group (vehicle); Comparison: ANP (A. nodosum) and USP (*U. pinnatifida*) polysaccharides	4 weeks HFD + STZ induction; 8 weeks treatment	HOMA-IR↓ (best among three seaweed polysaccharides tested)	[46]
*Fucus vesiculosus*	Fuc	→3)-α-L-Fucp-(1→4)-α-L-Fucp-(1→	Water-extracted	In vitro: DPPH radical scavenging assay; COX-1/COX-2 inhibition assays; hyaluronidase inhibition; DPP-IV inhibition; Cell-based: LPS-stimulated human U937 mononuclear cells	Enzyme assays: 0.01–100 µg/mL; DPP-IV: IC_50_ = 1.11 µg/mL; COX-2: IC_50_ = 4.3 µg/mL; hyaluronidase: IC_50_ = 2.9 µg/mL	DPP-IV: IC_50_ = 1.11 µg/mL; COX-2: IC_50_ = 4.3 µg/mL	Positive control: indomethacin (COX inhibitor); sitagliptin (DPP-IV inhibitor); Negative control: enzyme + substrate without fucoidan	Single-dose in vitro assay	DPP-IV↓ and consequent prolongation of endogenous incretin activity	[47]
*Gracilaria gracilis*	Glu, ManA, GlcA	____	Water extraction + ethanol precipitation + purification	In vivo: HFD- and streptozotocin (STZ)-induced T2DM rats	SPCs: 150, 300 mg/kg/day; STZ: 45 mg/kg i.p. (after HFD); Metformin: standard dose	300 mg/kg/day	Positive control (drug): metformin; Negative control: T2D control group (vehicle-treated); Normal control: non-diabetic rats	5 weeks of treatment after T2D induction	IRS-1-mediated insulin signaling↑, leading to GLUT4 expression↑ and improved glucose uptake	[48]
*Undaria pinnatifida*	Fuc, Gal, GlcA, Man;	→3)-α-L-Fucp-(1→4)-α-L-Fucp-(1→	Microwave-assisted extraction;	In vitro: α-glucosidase inhibition assay; insulin-resistant HepG2 cells (high glucose/insulin-induced) In vivo: HFD/STZ-induced hyperglycemic mice	In vitro: 0.01–0.1 mg/mL (Up-3, Up-4 strong inhibitory activity); HepG2: 0.1–1 mg/mL In vivo: 100, 200, 400 mg/kg/day; STZ: 60 mg/kg i.p.; Metformin: 200 mg/kg/day	In vitro: Up-3 and Up-4 significant at 0.05 mg/mL; In vivo: 400 mg/kg/day effective for FBG reduction	In vitro positive control: acarbose; In vivo positive control: metformin (200 mg/kg); Negative control: HFD/STZ model group	In vivo: 4 weeks HFD + STZ; 6 weeks Up-U treatment	α-glucosidase↓; postprandial blood glucose↓; FBG↓; insulin resistance↓; pancreas islet damage alleviated; hepatic steatosis↓	[49]
*Marine-derived chondroitin sulfate (shark cartilage)*	GlcA, GalNAc	→3)-β-D-GlcA-(1→4)-β-D-GalNAc-(1→	____	In vitro: porcine pancreatic α-amylase inhibition assay In vivo: STZ-induced diabetic	In vitro: 0.1–100 mg/mL (IC_50_: shark CS = 11.97 mg/mL; porcine CS = 14.42 mg/mL); In vivo: oral 200 mg/kg	In vitro: IC_50_ = 11.97 mg/mL (shark CS); In vivo: 200 mg/kg	Positive control: acarbose (in vitro enzyme inhibition); Negative control: enzyme + substrate without CS; In vivo: glucose challenge alone (vehicle)	In vitro: single-dose assay; In vivo: 2 h postprandial glucose monitoring	α-amylase activity↓; postprandial blood glucose↓; delayed starch hydrolysis	[50]
*Ecklonia maxima*	α-L-Fuc	α-(1→3), alternating α-(1→3)-α-(1→4) linkages	extracted by hot water	In vitro: α-amylase and α-glucosidase inhibition assay; Acarbose as reference standard	Fucoidan from *E. maxima*: 0.01–1.0 mg/mL; *F. vesiculosus* fucoidan and acarbose: same range for comparison	IC_50_ for α-glucosidase: 0.27–0.31 mg/mL (mixed-type inhibitor)	Positive control: acarbose (commercial α-glucosidase inhibitor); *F. vesiculosus* fucoidan (comparative standard); Negative control: enzyme + substrate without fucoidan	Single-dose in vitro assay; enzyme kinetics (Michaelis–Menten, Lineweaver–Burk analysis)	α-Glucosidase↓ (potent mixed-type inhibitor); minimal effect on α-amylase; selective glycemic control without significant GI side effects	[51]

Note: ↑ and ↓ indicate increased or decreased levels, expression, activity, or signaling, respectively, compared with the corresponding control group.

**Table 3:** Chemical characteristics and glucose-regulating activities of representative seaweed-derived bioactive compounds.

Compound/Fraction	Compound Class	Seaweed Source	Chemical Classification	MW (Da or kDa)	Key Structural Features
Dieckol	Polyphenol	*Eisenia bicyclis*	Phlorotannin (eckol-type)	742.5 Da	High hydroxyl density; high polymerization
Phlorofucofuroeckol A	Polyphenol	*Ecklonia stolonifera*	Phlorotannin	602.5 Da	Dibenzodioxin skeleton; multiple phenolic OH groups
7-Phloroeckol	Polyphenol	*Ecklonia cava*	Phlorotannin	478.4 Da	Polyhydroxylated aromatic rings
DPHC	Polyphenol	*Ishige okamurae*	Phlorotannin derivative	480.4 Da	Multiple hydroxyl substitutions
Fucoidan	Polysaccharide	*Undaria pinnatifida*	Sulfated polysaccharide	1–1000 kDa	Sulfation; branched structure
Alginate	Polysaccharide	*Brown algae*	Uronic acid polysaccharide	10–600 kDa	M/G block arrangement
Laminarin	Polysaccharide	*Laminaria japonica*	β-glucan	2–10 kDa	Linear glucan backbone
Ulvan	Polysaccharide	*Green algae*	Sulfated heteropolysaccharide	150–2000 kDa	Sulfation; uronic acids
Carrageenan	Polysaccharide	*Red algae*	Sulfated galactan	100–800 kDa	Sulfate-rich backbone

## Data Availability

The original contributions presented in this study are included in the article.
